# From Data to Decision Making: A Usability Study of the ESSY Whole Child Screener Data Reports

**DOI:** 10.3390/bs16071067

**Published:** 2026-06-29

**Authors:** Jessica B. Koslouski, Amy M. Briesch, Jacqueline M. Caemmerer, Sandra M. Chafouleas

**Affiliations:** 1 Department of Educational Psychology, University of Connecticut, Storrs, CT 06269, USA; jacqueline.caemmerer@uconn.edu (J.M.C.); sandra.chafouleas@uconn.edu (S.M.C.); 2 Bouvé College of Health Sciences, Northeastern University, Boston, MA 02115, USA; a.briesch@northeastern.edu

**Keywords:** whole child, universal screener, multi-tiered systems of support, data interpretation, usability

## Abstract

We are developing the Expanding Screening to Support Youth (ESSY) Whole Child screener to provide an efficient and comprehensive school-based screening instrument. Existing evidence suggests that teachers may have difficulty interpreting and using data reports, with implications for educational decision making. We used a sequential explanatory mixed methods design to evaluate three data report options for the ESSY Whole Child Screener. A U.S. sample of 285 teachers were randomized to one of three conditions: data report, data report + basic interpretation guide, and data report + expanded interpretation guide. Participants viewed a video recording of a mock data team reviewing data for a fictional student using the assigned materials. Participants then evaluated the usability of the assigned data report materials on six constructs of usability. Qualitative interviews were conducted with nine participants. Quantitative results indicate that teachers had similar perceptions of usability across conditions with the exception of the data report + basic interpretation guide reducing perceived need for additional professional development or consultation. Qualitative findings suggest similar perceptions of usability across conditions. We discuss implications for finalizing the instrumentation of the ESSY Screener, school-based data use, and measure development.

## 1. Introduction

At the foundation of an effective multi-tiered system of support (MTSS) is the use of universal screening. Within universal screening, all students are proactively assessed using brief measures to identify any signs of possible risk as early as possible and help focus subsequent intervention efforts. Schools routinely conduct these types of screenings in the areas of academics (e.g., reading, math) and physical health (e.g., vision, hearing), with screening for social, emotional, and behavioral (SEB) concerns being less common (Briesch et al., 2022a). Current screening efforts in schools tend to be limited, however, in their focus on one area of student functioning at a time, thereby leaving educators with an incomplete picture of student needs (Koslouski et al., 2024). Research makes clear that children’s learning, health, and development are deeply interconnected—academic struggles do not occur in isolation from SEB functioning or physical well-being (Comer et al., 2004; Darling-Hammond & Cook-Harvey, 2018). Therefore, to support the whole child, schools need screening tools that align with this reality. An integrated screening assessment—one that examines multiple domains of student functioning at once—would allow educators to more efficiently and accurately identify where a student needs support and ensure that the right resources are put in place across all areas of development.

In response to these concerns, we are developing the Expanding Screening to Support Youth (ESSY) Whole Child Screener to provide a more comprehensive view of the whole child in context. The ESSY Screener assesses both within-child strengths and concerns across five domains (i.e., Academic Skills, Behavior, Emotional Well-Being, Physical Health, Social Skills) as well as elements of the student’s school and community contexts across four domains (i.e., Access to Material Needs, Attendance, School Inclusion and Belonging, Social Support Outside of School). As one of the hallmarks of an effective screener is the feasibility of administration (Glover & Albers, 2007), the ESSY Screener utilizes a multiple-gated approach to balance both efficiency and comprehensiveness. At Gate 1, teachers rate all students in their classrooms across nine broad items corresponding to the aforementioned domains. If teacher ratings indicate concern for a particular student on a particular domain, the teacher then completes a series of items at Gate 2 designed to provide a more detailed assessment of that domain to help inform subsequent intervention efforts. If teacher ratings do not indicate a concern on any of the domains, screening for that student concludes after Gate 1.

Although the ESSY Screener was designed to capture multiple domains of student functioning within one relatively brief measure, one guiding concern in the development process has been how best to present the resultant data to maximize usability. Research has repeatedly shown that educators face significant challenges with data literacy, both in interpreting academic data (e.g., Pierce & Chick, 2013) and in translating these data into actionable intervention decisions (Jacobs et al., 2009). It is therefore unsurprising that many educators characterize data use as overwhelming or stressful (Schelling & Rubenstein, 2021) and report low levels of self-efficacy when it comes to data use (Dunn et al., 2013). Low self-efficacy may help to explain why conversations among school teams often stray from focus on the data at hand to external attributions (e.g., family background, student characteristics) that are outside of the control of educators (Little, 2012; Schildkamp & Datnow, 2022). These challenges are even further compounded when considering SEB domains, where educators have had fewer opportunities to develop confidence with screening data and report greater ambiguity in knowing how to respond to SEB data (Briesch et al., 2022b; Marsh & Kennedy, 2020).

For this reason, a core feature of the ESSY Screener is an integrated data report designed to translate screening results into actionable information for educators. The development of the data report was guided by established best-practice standards for how information should be organized, displayed, and communicated to end users through educational data reports (Rankin, 2016). Consistent with these standards, as well as findings from empirical studies on data report usability (e.g., Locke et al., 2023; Shivraj & Ketterlin-Geller, 2019), particular attention was paid to ensuring that visual elements supported efficient and accurate interpretation of results. Further, given that the ESSY Screener captures student functioning across nine distinct domains, a central goal was to produce a report that was both comprehensive and succinct. To meet this challenge, the development of the report incorporated input from potential end users (i.e., educators) to ensure that the final product was organized, interpretable, and directly useful for informing decisions about student support. Overall, educators responded positively to the data report, finding it well-formatted, sufficiently informative to support decision-making, and useful across multiple practical applications (Gertz et al., under review).

Although helpful in making screening data more accessible and usable for educators, data reports are not uncommon among screening measures. However, rather than incorrectly assuming that the data itself will indicate the appropriate next steps, it has been argued that additional supports are needed to guide educators in their data use (Spillane, 2012). It was for this reason, for example, that Todd et al. (2011) developed their structured problem-solving protocol for Team-Initiated Problem Solving, or TIPS. TIPS guides teams through a standardized process to (a) identify the problem, a goal, and potential solutions, as well as to (b) monitor implementation integrity and outcomes. Horner et al. (2018) found that those school teams that received TIPS training were more likely to describe their interventions as producing meaningful behavioral change than teams that did not receive TIPS training. This suggests that providing teams with a more defined structure for interacting with their data may serve to improve both team and student outcomes.

In response to documented concerns about effective data use in schools, we therefore developed two versions of a data interpretation guide (basic and expanded) to accompany the ESSY Screener data report (both versions of the guide will be further described in the Materials and Methods section). The ESSY Screener’s basic interpretation guide is designed to facilitate analysis and discussion of the results by orienting users to the data, asking about patterns and potential interventions, and developing progress monitoring plans. The expanded version of the ESSY Screener interpretation guide, on the other hand, aims to address some of the concerns that have been raised regarding narrowed focus on collected data. Although there is a substantial literature base on data-based decision making (DBDM; Ikemoto & Marsh, 2007), more recently scholars have critiqued this model as presuming data are objective and neutral, rather than deeply influenced by the circumstances in which they are collected, interpreted, and used (Dodman et al., 2021). Dodman et al. (2021) argue that educators’ beliefs about data, students, their own efficacy, and agency are key influences in data use practices. Our goal was to address some of these concerns within the expanded interpretation guide through priming and self-reflection activities (e.g., “we believe” statement about high expectations and growth mindset, a neutralizing routine to acknowledge and set aside any negative emotions) as well as a closing activity to reflect on how the data were used.

Throughout our development of the ESSY Whole Child Screener, we have maintained an explicit focus on understanding both the intended and unintended consequences of the instrumentation using a 10-step consequential validity-centered measure development (CVMD) framework (see Caemmerer et al., 2026a). Although both data interpretation guides were developed to aid data teams in using screening data to inform next steps for students, these guides will ultimately only be useful to the degree to which they are usable. Grubb and Young (2024) previously created a data interpretation guide to support team discussions of behavior screening data obtained using the Student Risk Screening Scale-Internalizing and Externalizing (SRSS-IE; Lane & Menzies, 2009). The data interpretation guide focused on identifying additional necessary data sources, trends and patterns in the data, potential instructional strategies to meet student needs, and progress monitoring approaches. In follow-up focus groups, educators emphasized that the structured questions prompted conversations and new insights about students’ needs, but some participants reported feeling overwhelmed by the time and effort needed to fully grapple with the data using the interpretation guide. Datnow and Park (2018) reported some educators describing highly productive data team meetings reviewing data using an interpretation guide. Others, however, expressed relief when finishing data team discussions guided by an interpretation guide, suggesting it was perceived as a task to complete rather than a tool to enable new insights and thoughtful planning. This suggests that the use of such interpretation guides needs to be carefully examined for intended and unintended consequences, as well as perceived usability.

Given both the more comprehensive nature of the ESSY Screener as well as the availability of both basic and expanded interpretation guides, exploration of the usability of these resources represents a critical next step in the development and validation of the ESSY Screener instrumentation. As such, this study sought to compare the perceived usability of three different versions of the ESSY data report: (1) data report, (2) data report + basic interpretation guide, and (3) data report + expanded interpretation guide. Our research questions were: (1) How do teachers perceive the usability of different data report versions for the ESSY Whole Child Screener? (2) Does previous screening or data team experience affect teachers’ perceptions of usability of different data report versions of the ESSY Whole Child Screener? We hypothesized that the conditions including a data interpretation guide (basic or expanded) would be rated as more usable than the data report alone due to the additional scaffolding and support provided. We also hypothesized that teachers with previous screening and data teams experience would rate the usability of the data reports higher given their familiarity with these procedures. This information is important for finalizing the instrumentation of the ESSY Whole Child Screener and has implications for supporting data interpretation and use in schools.

## 2. Materials and Methods

We used a sequential explanatory mixed methods design (Creswell & Plano Clark, 2017) to investigate the usability of three different versions of the ESSY data report. The three versions were the (1) ESSY data report, (2) ESSY data report + basic interpretation guide, and (3) ESSY data report + expanded interpretation guide. First, we collected quantitative survey data regarding the usability of the three different versions. Then, we collected qualitative interview data to deepen our understanding of the quantitative data, with an emphasis on understanding participants’ perceptions of the usability of the data report materials, which aspects were perceived as helpful, and what could be improved. In both phases of the study, we used fictional student data reports to standardize the materials across participants. In each phase, we randomized participants to one of the three data reports, allowing us to compare perceptions of usability across conditions. The study was approved by the Northeastern University Institutional Review Board.

### 2.1. Vignette Materials

#### 2.1.1. The ESSY Whole Child Data Report

The ESSY screening report has four sections summarizing individual student-level data. Section 1 briefly describes the ESSY Whole Child Screener, defines the child and contextual domains assessed by the screener, and summarizes the gating procedure. This section is the same across all students and does not contain any student-level information. The next section summarizes the teacher’s Gate 1 responses. Child and Contextual domains are listed in one of five color-coded categories, ranging from *Area of Substantial Strength* to *Area of Substantial Concern*. These are drawn from the teacher’s ratings during Gate 1 of the measure. Section 2 also indicates whether the teacher’s ratings suggest the student and teacher have a positive or negative relationship. Section 3 includes Gate 2 responses for any Child domains indicated as an area of concern at Gate 1. Teacher ratings are organized into three color-coded categories: *Strengths to Maintain*, *Areas to Monitor*, and *Concerns for Follow-Up*. The stem of each item is listed in its respective column based on how the teacher rated the student. Section 4 does the same for any Contextual domains rated as an area of concern at Gate 1. Demographic information about the student (i.e., name, race/ethnicity, gender, special education status, grade, teacher) and the date the screener was completed are presented at the top of Section 2, Section 3 and Section 4.

In the present study, the ESSY data report was created for two fictional students: one for the quantitative portion of the study and another for the qualitative portion of the study. For the *quantitative* portion of the study, the student was rated as having areas of substantial strength in the Child domains of Physical Health and Social Skills. Emotional Well-being was rated as an area of some concern. Academic Skills and Behavior were rated as areas of substantial concern. In the Contextual domains, School Inclusion and Belonging as well as Social Support Outside of School were rated as areas of some strength. Attendance was rated as neither a strength nor concern, and Access to Material Needs was rated as an area of some concern. As such, Section 3 of the data report was completed for Academic Skills, Behavior, and Emotional Well-being. Section 4 of the data report was completed for Access to Material Needs.

A different fictional student’s data report was created for the *qualitative* portion of the study. For the Child domains, the student was rated as having an area of some strength in Physical Health. Behavior was rated as neither an area of strength nor concern. Academic Skills and Social Skills were rated as areas of some concern and Emotional Well-Being was rated as an area of substantial concern. In the Contextual domains, Access to Material Needs and Attendance were rated neither strengths nor concerns. School Inclusion and Belonging was rated as an area of some concern and Social Support Outside of School was rated as an area of substantial concern. Consequently, Section 3 of the data report was completed for Academic Skills, Emotional Well-being, and Social Skills. Section 4 of the data report was completed for School Inclusion and Belonging and Social Support Outside of School.

#### 2.1.2. ESSY Data Interpretation Guides

As previously described, we have developed two ESSY data interpretation guides (basic and expanded versions) as potential accompaniments to the ESSY data report. These are intended to scaffold the data interpretation process for users, improving opportunities for data use and meaningful educational decision making. The basic version includes nine questions organized into three sections: integrity and trustworthiness of data; identifying primary strengths, concerns, and potential explanations; and next steps. The expanded version includes 15 questions, including the nine questions from the basic version. The expanded version also includes an opening and closing routine. The opening routine includes a self-check and “we believe” statement to encourage a strengths-based mindset for the data review. The closing routine includes three questions for users to briefly reflect on the data review process.

#### 2.1.3. Videos of Mock Data Team Discussions

For the quantitative portion of the study, videos of mock data team discussions were created to build participant’s familiarity with the ESSY data reports and their intended use. Three videos were created. The first video showed a mock data team interpreting the information provided on the ESSY data report for the fictional student described above. The second video showed the team interpreting the information using the ESSY data report + basic interpretation guide. In the third video, the team used the ESSY data report + expanded interpretation guide. All three members of the data team have worked in schools and the team leader was previously an elementary school data team leader. The research team created and used scripts for these videos, ensuring that each condition used only the provided materials. The scripts were checked to ensure questions from the interpretation guides were only verbalized in relevant conditions. The data report used in all three videos was the same, making the addition of the basic or expanded interpretation guide the only difference in materials. Each video used split screen editing to show the data team discussing the information while also showing a zoomed in version of the data report and relevant interpretation guide, as applicable. This allowed participants to see and hear the data team discussion while also viewing the materials for themselves. Each video was approximately seven minutes in length and edited by a graduate research assistant with professional video editing experience.

### 2.2. Quantitative Recruitment and Participants

To recruit a broad sample of third, fourth, and fifth grade teachers, participants were recruited using a Qualtrics panel. Inclusion criteria included currently teaching third, fourth, or fifth grade in the United States. Screening questions were used at the beginning of the survey to determine eligibility. In total, 285 participants enrolled in the study. Participant demographics are shown in Table 1. The sample was 81% female. Fifty-six percent reported their highest level of education as a bachelor’s degree. Close to 40% had earned a master’s degree and the remaining 4% held a professional or doctoral degree. Eighty percent were general education teachers and 20% were special education teachers. Similar proportions of participants taught 3rd and 4th grade, with slightly less teaching 5th grade.

### 2.3. Quantitative Measures

#### 2.3.1. Usage Rating Profile—Assessment

The Usage Rating Profile—Assessment (URP-A; Chafouleas et al., 2012) evaluates teachers’ perceived usability of school-based assessments. The URP-A assesses six constructs of usability: acceptability, feasibility, understanding, system climate, home-school collaboration, and system support. Consistent with prior abbreviated versions of the URP (e.g., Ci3T-URP, Buckman et al., 2024), one item from each construct was asked to assess the perceived usability of the assigned ESSY data report (see Table 2). We modified the wording of the items to indicate our study’s focus on the screening report reviewed in the recorded data team meeting. Items were rated on a six-point scale ranging from strongly disagree to strongly agree. Higher scores on Acceptability, Feasibility, Understanding, System Climate, and Home-School Collaboration indicate higher usability. Due to its wording (see Table 2), a lower score on System Support indicates higher usability. A Total Usability score can be calculated by reverse scoring System Support and then summing the six scores. URP-A scores have been used to compare the usability of different school-based assessments and have demonstrated acceptable to high internal consistency (α = 0.63–0.90; Miller et al., 2014).

#### 2.3.2. Demographics

Participants were asked to provide their current teaching role, grade level(s) they currently teach, years of teaching experience, and highest level of education. They were also asked whether they had previously completed school-based screening measures or participated on a school-based data team. Finally, participants were asked their gender, ethnicity, and region of the United States.

### 2.4. Quantitative Data Collection Procedure

Qualtrics was programmed to randomize participants to one of three conditions: (1) data report, (2) data report + basic interpretation guide, or (3) data report + expanded interpretation guide. The sample contained 97 participants assigned to the data report condition, 89 participants assigned to the data report + basic interpretation guide condition, and 99 participants assigned to the data report + expanded interpretation guide. Within each condition, participants first watched a seven-minute video in which a data team used the materials in the assigned condition to interpret data for a fictional student. Then, participants rated the usability of the data report materials used in their condition. Participants also provided demographic information about themselves. Qualtrics compensated participants for their participation.

### 2.5. Quantitative Data Analysis

The six usability scores (Acceptability, Feasibility, Understanding, System Climate, Home-School Collaboration, System Support) were averaged across participants within each condition. Next, we calculated a Total Usability score for each participant by reverse scoring System Support and summing the six scores. We then performed ANCOVAs to compare the effect of data report condition on usability scores. The two binary demographic items about previous experience completing screeners and previous experience participating on school-based data teams were included as covariates. Where scores differed, post hoc testing using the Bonferroni correction was used to determine which data report conditions reported significantly different usability scores. Effect size for partial eta square were interpreted as small (0.01–0.05), medium (0.06–0.13), or large (≤0.14; Cohen, 1988). All analyses were conducted in IBM SPSS version 30.

### 2.6. Qualitative Recruitment and Participants

Qualitative participants were recruited in two ways. First, participants were recruited through the Qualtrics panel described above by asking if they would like to participate in a follow-up interview. This strategy yielded three participants for the qualitative phase of the study. To expand recruitment, teachers from the study authors’ professional networks were contacted directly via email to invite them to participate in the study. An additional six participants were recruited in this way. Across recruitment methods, eligibility criteria included being at least 18 years of age, comfortable completing the interview in English, currently a classroom or special education teacher, and currently teaching students in grades 3–5 in the United States.

Nine educators participated in qualitative interviews. Most were general education classroom teachers (*n* = 8), female (*n* = 8), White (*n* = 7), and taught in the Northeastern United States (*n* = 8). Participant characteristics are shown in Table 3.

### 2.7. Qualitative Data Collection Procedure

Prior to interviewing participants, two graduate assistants participated in a one-hour training session led by the first author. This training included a review of qualitative interviewing techniques as well as guided practice with the semi-structured interview protocol. Prior to the interview, participants provided informed consent and completed the same demographic form used in the quantitative study (described above). Each participant was assigned to one of the three data report conditions based on time of enrollment (e.g., the first participant was assigned to the data report condition, the second participant was assigned to the data report + basic interpretation guide condition, and so on). Participants were then interviewed by one of two graduate research assistants using teleconferencing software. Due to concerns related to confidentiality of responses, we could not link quantitative and qualitative data across the two phases of the study. Therefore, we do not know which condition the three qualitative participants recruited through the Qualtrics panel were assigned to in the quantitative portion of the study.

A semi-structured interview protocol was used to facilitate the interview. At the start of the interview, participants were shown a data report for a fictional student based on their assigned condition. To gain familiarity using the report, participants were first asked to “think aloud” (Nielsen, 1994) as they reviewed the report and to share their perspectives on the fictional student case. Then, participants were asked about various aspects of the report’s usability. They were asked one open-ended question about each aspect of usability: acceptability, feasibility, understanding, system climate, home-school collaboration, and system support. Interviews lasted 32–59 min (*M* = 45 min) and participants were given a $50 gift card in appreciation for their time. All interviews were audio recorded and transcribed verbatim using Otter.ai. Transcripts were then de-identified and verified by trained graduate assistants.

### 2.8. Qualitative Data Analysis

We used framework analysis (Gale et al., 2013) to investigate teachers’ perceptions of the usability of their assigned ESSY data report. Before coding, a spreadsheet was developed to guide the process. Based on the research question, columns included codes representing each aspect of usability (i.e., acceptability, feasibility, understanding, system climate, home-school collaboration, system support). Then, each of the nine transcripts was assigned a row, organized by condition. The spreadsheet was then duplicated to allow for independent coding. The first author and two trained graduate assistants read each transcript line-by-line, placing participant comments related to each code in the appropriate cell of the spreadsheet (participant x code). The first author coded all nine transcripts. Each of the graduate assistants coded 4–5 transcripts. All transcripts were double coded. The spreadsheets were then compared across coders to develop a consensus spreadsheet. Any cells with different data were discussed and consensus regarding inclusion and placement was reached. Once the consensus spreadsheet was created, the qualitative coding team explored patterns within and across participants, as well as by condition.

### 2.9. Mixed Methods Analysis

After the quantitative and qualitative data were analyzed and interpreted separately, they were organized into a joint display (Guetterman et al., 2015) to assess areas of similarity or divergence across the two types of data.

## 3. Results

### 3.1. Quantitative Results

Descriptive statistics for the usability scores across the three data report conditions are presented in Table 4. The mean scores for Acceptability, Feasibility, Understanding, System Climate, and Home-School Collaboration all fell between 4.6 and 5.1 (4 = slightly agree, 5 = agree), suggesting participants agreed they wanted to use the screening report in practice, understood how to do so, the resources (e.g., time) required would be reasonable, their system would be supportive of such a screening process, and they would collaborate with families in doing so. As discussed in the Methods, a lower score on System Support indicates higher usability. System Support scores fell between 3.7 and 4.5 (3 = slightly disagree, 4 = slightly agree), suggesting that participants felt they might need some additional supports (i.e., consultation or professional training) to use the screening report. Internal consistency reliability of the total usability composite score was adequate (Ω = 0.80, α = 0.54).

Scores were approximately normally distributed (absolute skewness < 1.51 and kurtosis ≤ 2.76). Levene’s Test of Equality of Error was not significant for any of the dependent variables (*p* = 0.06–0.99). Multicollinearity was not a concern. The correlation between previous screening and data team experience was statistically significant, but small (*r* = 0.27, *p* < 0.001), and the correlations between the two covariates and data report conditions were not statistically significant (*p* = 0.21 and 0.36). There were no missing data on the usability scores or data report condition variables. For the purposes of the ANCOVAs, “not sure” responses on the previous screening and data team experience covariates were coded as missing, which resulted in 2.9 to 3.5% missingness on the covariates (see Table 1).

Correlations between Acceptability, Feasibility, Understanding, System Climate, and Home-School Collaboration were statistically significant and ranged from small to moderate sized (*r* ranged from 0.27 between Acceptability and Home-School Collaboration to 0.52 between Feasibility and System Climate). System Support had relatively lower correlations with the other five usability domains (*r* = 0.23 with Acceptability and 0.27 with Home-School Collaboration); some were too small to be practically meaningful (*r* = −0.12 with Understanding and 0.15 with Feasibility) and the correlation between System Support and System Climate was not significant (*p* = 0.76).

The ANCOVA omnibus tests were not statistically significant for teachers’ perceptions of Acceptability (F(4, 280) = 2.22, *p* = 0.07), Feasibility (F(4, 280) = 0.03, *p* = 1.00), Understanding (F(4, 280) = 0.66, *p* = 0.62), System Climate (F(4, 280) = 0.14, *p* = 0.97), and Home-School Collaboration (F(4, 280) = 0.67, *p* = 0.61). This indicates that teachers had similar perceptions of usability in these five areas across the three different versions of the data report. Furthermore, the omnibus test for Total Usability was significant (F(4, 264) = 3.14, *p* = 0.02), but there were no significant differences across data report conditions (F(2, 264) = 0.10, *p* = 0.91). There was a significant main effect of previous experience participating on data teams (F(1, 264) = 6.08, *p* = 0.01), with a small effect size (η^2^p = 0.02). Teachers with previous experience on data teams reported higher total usability scores than those without previous data team experience.

The ANCOVA omnibus test was statistically significant for System Support (F(2, 280) = 2.77, *p* = 0.03). There were significant differences across at least two data report conditions, but there were no significant differences based on teacher’s experience completing school-based screeners or participating on school-based data teams. Post hoc testing using the Bonferroni correction indicated that the mean value of System Support was significantly different between the data report and data report + basic interpretation guide conditions (*p* = 0.01, 95% C.I. for difference = 0.15, 1.14). The effect size was small (η^2^p = 0.04). This indicates that participants assigned to the data report + basic interpretation guide were significantly less likely than those assigned to the data report condition to endorse needing professional development or consultation to use the screening report (see Table 4). Neither condition differed significantly from the data report + expanded interpretation guide.

### 3.2. Qualitative Findings

#### 3.2.1. Data Report

Participants who viewed only the ESSY data report perceived it to be usable. They reported that it was well laid out, easy to understand, and visually appealing. Participant 301 reported, “I like the color coding, it’s clear. The fonts are easy to read. […] Some of the bolded areas are great because it really draws your attention to it.” Participant 307 shared “Everything I’m reading is understandable and easy to understand and grasp. […] Things are very clear and presented well.” Participants expressed that the report was consistent with procedures in their schools (e.g., other rating scales, MTSS) and would be well-received by their peers so long as the initial measure completion took a manageable amount of time. Perspectives were mixed on home-school collaboration, with one participant saying that home-school collaboration would not be needed to use the screener but others stating that family input might be needed to accurately answer some of the contextual items. Participant 304 requested that the item stems be presented in a more concise way (“it became overwhelming to keep reading *sometimes*” [the Likert response option selected for these Gate 2 items]) and Participant 307 wanted to see numbers associated with the ratings. They explained, “I am missing actual numbers. So, was this on a scale of zero to 10? Was it zero to 100?” All three participants indicated that they would collaborate with colleagues to determine next steps and Participant 307 suggested the report offer recommended next steps.

#### 3.2.2. Data Report + Basic Interpretation Guide

Participants who viewed the data report and basic interpretation guide perceived it to be usable. They appreciated the inclusion of student strengths and commented that the organization and color coding made interpretation straightforward. Participant 308 commented that the first section, which introduced the domains assessed by the screener, was a helpful orientation to the report. Participant 302 appreciated the organization of Gate 1 and Gate 2 information. They remarked, “[The Gate 1 section] gave you an overview of everything, […] but the next section really kind of went into the nitty gritty.” Participant 308 commented, “I think the wording is very succinct, the way that it paints the picture.” Participants expressed that they would be able to use the screener without additional professional development, but suggested that a user video or guide could be a helpful resource. Participants 302 and 308 said that they might gather some information from families related to contextual aspects of each student’s life, which might be done during parent-teacher conferences or through a short survey sent via email. Participant 305 remarked that the data report would be a helpful tool for sharing information as students transitioned to the next grade level. Participants said they had the necessary skills to interpret the data, but would likely collaborate with colleagues to determine appropriate interventions. Participant 305 suggested that recommended interventions could be added to the report. Whereas Participants 305 and 308 said the screener aligned with their system’s climate, Participant 302 said that their school did not have an existing data team, which would make universal screening more challenging. Participant 302 was interested in using it for their own classroom of students. They also appreciated the lack of numbers used in the data report. Participant 302 explained, “I actually like that there’s no numbers. […] I feel like there’s no comparison. These are her strengths, she struggles with this, she’s okay in this. […] But I feel like it doesn’t compare with someone else.”

#### 3.2.3. Data Report + Expanded Interpretation Guide

Participants who viewed the data report and expanded interpretation guide perceived it as usable. They expressed appreciation for the inclusion of student strengths and remarked that the color coding and icons assisted with their interpretation. Participant 303 commented, “The icons, the images, help with just being able to identify the information and it makes it easy to understand what we’re looking at.” Participants also expressed appreciation for the thoroughness of the measure. Participant 309 said that the report would be very helpful to their school’s child study team, which discussed students who teachers were concerned about. Participant 309 explained, “This screener would be excellent to be able to use and then bring the results to the child study team. It’s an excellent, very thorough document.” Participant 303 felt that the report would be a useful communication tool as students transitioned to the next grade level. Participants felt that teachers would be able to use the screener with minimal professional development and suggested that a brief session dedicated to introducing the screener would be enough for successful use. Participant 309 said that they would inform families if they were going to be meeting with a team to review data about a specific student. Participant 303 shared that collaboration with families would be helpful for completing contextual items.

### 3.3. Mixed Methods Interpretation

A summary of the quantitative results and qualitative findings were organized into a joint display, which is shown in Table 5. Across both types of data, there are few differences in perceptions of usability based on participant condition. In many cases, the qualitative data provides potential explanations for the quantitative data. For example, in the qualitative data, participants expressed appreciation for the data report’s inclusion of student strengths and strong visual layout, which provides some insight into participants’ endorsement of the Acceptability of the measure within the quantitative data. Furthermore, in the quantitative data, participants expressed that they would communicate and collaborate with families to use the measure. In the qualitative data, participants explained that they would communicate with families to gather information about contextual aspects of students’ lives and also to be transparent about any team discussions related to supporting the student.

## 4. Discussion

Existing research suggests that data literacy varies among teachers, which can reduce opportunities for efficient and effective data use. Within the ESSY Screener instrumentation, we therefore aimed to address limitations of existing universal screening tools through the inclusion of both a data report and two potential data interpretation guides. The basic and expanded interpretation guides offered varied levels of scaffolding and guided reflection to accompany the data report. Although more scaffolding might seem justified given concerns related to teachers’ data literacy, time is a known barrier to data interpretation and use (Grubb & Young, 2024; Koslouski et al., 2026). Consistent with the CVMD framework (Caemmerer et al., 2026a), this study therefore focused on exploring both the usability and potential consequences of use to inform any necessary modifications to the instrumentation. Specifically, within this mixed methods study, we used an explanatory sequential design (Creswell & Plano Clark, 2017) to investigate the perceived usability of three different versions of the ESSY data report.

Contrary to our first hypothesis, both the quantitative and qualitative data showed few differences in the perceived usability of the three versions of the ESSY data report. Across data sources, the Acceptability of the three versions of the ESSY data report was rated favorably. Qualitative data suggests this was due to the inclusion of student strengths, clear identification of areas in need of support, and visual organization of the report. Feasibility was also rated favorably, but participants raised questions in the qualitative interviews regarding the amount of time needed for teachers to complete the screener itself. This information is included in the ESSY User Manual (approximately 1–6 min per student depending on how many areas of concern are endorsed at Gate 1; Chafouleas et al., 2025) and will be important information for schools considering use of the measure. Across both the quantitative and qualitative data, teachers endorsed having the knowledge and skills needed to use the measure. Some qualitative participants said they would consult with colleagues regarding certain concerns (e.g., school psychologist regarding emotional well-being). This aligns with expected practices of teaming and collaboration to address student needs in schools. Finally, both qualitative and quantitative participants said that their schools would be supportive of using such a measure. Qualitative participants elaborated that the measure aligned with their work related to MTSS, universal SEB screening, and collaboration regarding student development.

Across data sources, teachers said they would collaborate with families to use the measure. Qualitative participants explained that they would collaborate with families to gather information about the contextual aspects of students’ lives (i.e., assets and barriers outside of school). They also said that they would notify families if a school-based team was going to discuss their child and to maintain collaboration and communication with families regarding any suggested supports. This finding aligns with Koslouski et al.’s (2024) previous finding that participants were unsure that teachers would be able to rate the ESSY Screener’s contextual items accurately. Future research should investigate concordance between teacher and student or family caregiver ratings to determine whether a multi-informant approach is needed, especially for the contextual items.

The quantitative data indicated that teachers assigned to the data report + basic interpretation guide endorsed significantly less need for professional development or consultation than those assigned to the data report only. The difference only had a small effect size, and therefore should be interpreted with caution. The result suggests, however, that the basic interpretation guide may be providing the scaffolding intended to support teachers’ interpretation of the data. In the qualitative data, participants endorsed needing minimal professional development or consultation to use the materials. Across conditions, participants felt that an introductory video, guide, or short training session would be sufficient for effective use. Future research will need to examine varied professional development needs or the effects of the interpretation guides on effective data use. Our second hypothesis was partially supported. Teachers with previous data team experience reported higher total usability scores than those without previous data team experience. This was not the case, however, for teachers with previous screening experience. It is likely that those with previous data team experience have more familiarity with interpreting data reports. This may not be the case for teachers who have filled out school-based screeners.

### 4.1. Implications for Finalizing the ESSY Screener’s Instrumentation

This study informs next steps in finalizing the ESSY Screener’s instrumentation. First, although the high levels of reported usability within this study suggest that the ESSY data report is perceived as usable, some participants did recommend developing a short video or user guide to inform use. A short instructional video could be used to supplement the training and implementation materials already developed and may provide users with a more accessible overview of procedures. Second, although prior studies have suggested that some educators may perceive use of data interpretation guides as burdensome (e.g., Datnow & Park, 2018; Grubb & Young, 2024), similar perceptions of usability were identified across conditions in the current study. This suggests that participants did not find the additional interpretation guides to be too burdensome. In a related study with school-based data teams, we found that teams preferred an optional interpretation guide to accompany the ESSY data report (Koslouski et al., 2026). They were interested in having access to the expanded guide, but planned to condense the questions for their setting and needs. Teams suggested varied use of the guide based on available time, case complexity, and team skills and norms. Together, these findings suggest that an optional interpretation guide alongside the ESSY data report may be the optimal instrumentation, allowing for differentiated scaffolding across users and settings.

### 4.2. Implications for Measure Development

This study also has implications for measure developers. Although the usability of assessment reports is not always evaluated with end users prior to publication (Caemmerer et al., 2026a), this study illustrates how this information can inform data report revisions and accompanying instrumentation (e.g., videos, user guides) to optimize data interpretation and use. Participants in the present study appreciated the organization, color coding, and icons used in the ESSY data report. This aligns with Rankin’s (2016) guidelines as well as findings by Locke et al. (2023), which emphasize the importance of focusing the reader’s attention by highlighting specific information and limiting the number of graphical elements. Shivraj and Ketterlin-Geller (2019) emphasize the importance of the report layout and the clarity of data summaries. As described elsewhere (Caemmerer et al., 2026a), we encourage measure developers to evaluate the perceived usability of their instrumentation throughout the development process to drive revisions and optimal data use.

### 4.3. Limitations and Future Directions

It is important to note the limitations of this study. First, to assess usability during the development process, the data report used in this study included the hypothesized constructs of the ESSY Screener. Exploratory and confirmatory factor analyses have since informed construct revisions (Caemmerer et al., 2026b), necessitating some revisions to the ESSY data report. That said, these revisions did not change the visual “feel” or elements used in the report. Next, although use of a Qualtrics sample allowed us to recruit a geographically diverse sample of U.S. teachers, Qualtrics programming encourages brevity in survey measures. Because the video was seven minutes long, we elected to use a single item to assess each usability construct. Thus, the usability constructs were more narrowly defined than their respective multiple-item subscales. These narrower usability constructs may have limited our ability to detect more nuanced differences between the data report conditions. Our qualitative data is limited by a small sample spread across conditions. Although participants expressed similar perceptions of usability across conditions, they primarily focused their responses on the data report itself. Those exposed to the basic or expanded interpretation guide rarely provided feedback on these, limiting our ability to develop conclusions about the usability of the interpretation guides themselves. Due to privacy concerns, we could not link quantitative and qualitative data. It is possible that the assigned condition in the quantitative survey influenced participants’ responses in the qualitative interview, but the “think-aloud” procedure was used to orient qualitative participants to the new materials. No participant brought up comparisons to the quantitative portion of the study.

Future research should investigate how the use of numbers versus descriptive items affects educational decision making. Although numbers allow for the use of cut scores, they may preclude the identification of student strengths, root causes, or alternative explanations. Future research should also examine the professional development or scaffolds that improve teachers’ data literacy, interpretation, and use. It will also be important to assess teachers’ perceptions of the usability of the ESSY Screener in applied use with the students in their classrooms. Moreover, examining the consequential validity (i.e., positive and negative consequences of use) when the ESSY Whole Child Screener is used in authentic screening contexts (i.e., Step 10 of the CVMD process; Caemmerer et al., 2026a) will provide insight into decisions made with the data and consequences for students, staff, and families.

## Figures and Tables

**Table 1:** Quantitative Participant Demographics (*N* = 285).

Participant Characteristics	*n*	%
Current Role		
Classroom Teacher	228	80.0%
Special Education Teacher	57	20.0%
Grade Level ^a^		
3rd	137	48.1%
4th	139	48.8%
5th	110	38.6%
Years in Education		
1–5	48	16.8%
6–10	72	25.3%
11–15	40	14.0%
16–20	44	15.4%
21–24	19	6.7%
25+	62	21.8%
Experience Completing School-Based Screening Measures		
Yes	236	82.8%
No	38	13.3%
Not Sure	11	3.9%
Experience Participating on a School-Based Data Team		
Yes	219	76.8%
No	59	20.7%
Not Sure	7	2.5%
Role on This Team		
Team Leader	35	16.0%
Participant Related to Specific Student(s)/Case(s)	93	42.5%
Ongoing Participant who Regularly Attends Team Meetings	88	40.2%
Other	3	1.4%
Gender		
Female	231	81.1%
Male	53	18.6%
Other	1	0.4%
Hispanic/Latino/Spanish Origin		
Yes	36	12.6%
No	248	87.0%
Prefer Not to Say	1	0.4%
Highest Degree		
Bachelor’s Degree	160	56.1%
Master’s Degree	112	39.3%
Doctorate	4	1.4%
Professional Degree	9	3.2%
Region of the United States		
Midwest	68	23.9%
Northwest	69	24.2%
South	99	34.7%
West	49	17.2%

^a^ *N* = 285, some teachers might have taught multiple grade levels.

**Table 2:** Usage Rating Profile—Assessment, Single Items.

Construct	Item
Acceptability	I would be interested in using the screening report reviewed during this data team meeting.
Feasibility	The time and other resources needed to use the screening report reviewed during this data team meeting would be reasonable.
Understanding	I would have the understanding and skills needed to use the screening report reviewed during this data team meeting.
System Climate	The system that I work in would be supportive of using the screening report (i.e., data collection through review) reviewed during this data team meeting.
Home-School Collaboration	Collaboration and communication with a student’s family would be needed to use the screening report reviewed during this data team meeting.
System Support *	I would need additional supports (i.e., consultation or professional training) in order to use the screening report reviewed during this data team meeting.

* Lower scores indicate higher usability.

**Table 3:** Qualitative Sample Participant Characteristics.

Participant #	Condition ^a^	Current Role	Grade Levels Taught	Years Working in Education	Gender	Race/ Ethnicity	Region (U.S.)
301	1	Special Education Teacher	4, 5	14	Female	White	Northeast
302	2	Classroom Teacher	5	8	Female	Latina	Northeast
303	3	Classroom Teacher	3	10	Female	Black	South
304	1	Classroom Teacher	5	25	Female	White	Northeast
305	2	Classroom Teacher	5	5	Female	White	Northeast
306	3	Classroom Teacher	5	25+	Female	White	Northeast
307	1	Classroom Teacher	5	25+	Male	White	Northeast
308	2	Classroom Teacher	4	1	Female	White	Northeast
309	3	Classroom Teacher	3	25	Female	White	Northeast

^a^ Condition 1 = data report; Condition 2 = data report + basic interpretation guide; Condition 3 = data report + expanded interpretation guide.

**Table 4:** Descriptive Statistics and Analysis of Covariance Results.

Usability Construct	Data Report (M, SD)	Data Report + Interpretation Guide (M, SD)	Data Report + Expanded Interpretation Guide (M, SD)	F	*p*
Acceptability	4.92 (1.04)	4.61 (1.10)	4.86 (1.08)	2.22	0.07
Feasibility	4.78 (1.11)	4.76 (0.89)	4.77 (1.03)	0.03	1.00
Understanding	5.08 (0.86)	4.91 (0.85)	4.94 (0.92)	0.66	0.62
System Climate	4.84 (1.11)	4.89 (0.82)	4.80 (1.11)	0.14	0.97
Home-School Collaboration	5.04 (1.07)	4.92 (1.13)	4.96 (1.09)	0.67	0.61
System Support ^a^	4.42 (1.32)	3.79 (1.43)	4.00 (1.45)	2.77 *	0.03 *
Total Usability ^b^	27.24 (3.57)	27.30 (3.09)	27.32 (4.10)	3.14 *	0.02 *

^a^ A lower system support score indicates greater usability. ^b^ System climate was reverse coded when calculating the total usability score so that higher scores of total usability indicate greater perceived usability. * Denotes statistically significant omnibus tests (*p* < 0.05).

**Table 5:** Joint Display of Qualitative and Quantitative Findings.

Usability Construct	Summary of Quantitative Results	Summary of Qualitative Findings
Acceptability	Across conditions, participants agreed they would be interested in using the data report.	Across conditions, participants appreciated the visual presentation and organization of the report. They also appreciated the inclusion of student strengths and clear communication of areas in need of support.
Feasibility	Across conditions, participants agreed the resources required to use the screening report would be reasonable.	Across conditions, participants asked about the amount of time required to complete the screener.
Understanding	Across conditions, participants agreed they had the knowledge and skills needed to use the data report.	Across conditions, participants expressed having the knowledge and skills needed to use the report.
System Climate	Across conditions, participants agreed the system they worked in would be supportive of using the screening report.	Across conditions, participants noted their system would be supportive of using the screening report. In one instance where the school did not have an existing data team, the participant felt it could be used by individual teachers.
Home-School Collaboration	Across conditions, participants agreed they would communicate and collaborate with families to use the screener.	Across conditions, participants said they would communicate with families to gather information about contextual factors in students’ lives and to alert them to any next steps (e.g., data team meeting, intervention) suggested by the data.
System Support	Participants assigned to the data report + basic interpretation guide reported less need for additional professional development or consultation than those assigned to the data report condition, suggesting the data report + basic interpretation guide may reduce the need for additional support.	Across conditions, participants said that minimal to no training would be needed. At most, participants felt that an introductory video, guide, or short training session would be sufficient for effective use.

## Data Availability

The original data presented in the study are openly available in OPENICPSR at https://doi.org/10.3886/E248040V1.

## Abbreviations

The following abbreviations are used in this manuscript:

ESSY	Expanding Screening to Support Youth
MTSS	Multi-Tiered Systems of Support
SEB	Social, emotional, and behavioral
